# Bioinformatics Analysis of circRNA Expression and Construction of “circRNA-miRNA-mRNA” Competing Endogenous RNAs Networks in Bipolar Disorder Patients

**DOI:** 10.3389/fgene.2021.718976

**Published:** 2021-08-04

**Authors:** Yonghui Fu, Wenfeng He, Chaoxiong Zhou, Xia Fu, Qigen Wan, Ling He, Bo Wei

**Affiliations:** ^1^Department of Psychiatry, Jiangxi Mental Hospital, Nanchang, China; ^2^Jiangxi Key Laboratory of Molecular Medicine, The Second Affiliated Hospital of Nanchang University, Nanchang, China; ^3^Department of Cardiovascular Medicine, The Second Affiliated Hospital of Nanchang University, Nanchang, China

**Keywords:** bipolar disorder, circular RNA, competitive endogenous RNA, mRNA, High throughput sequencing

## Abstract

Bipolar disorder (BD) is a severe mood disorder disease in China, and its underlying pathogenesis remains unknown. Circular RNAs (circRNAs) have been reported to play a key role in mental disorders and can be used as competitive endogenous RNAs (ceRNAs). However, little is known about the correlation of circRNAs with BD. In this study, Deep RNA sequencing was used to identify differentially expressed circRNAs (DE-circRNAs) and differentially expressed mRNAs (DE-mRNAs) between BD patients and a control group. Real-time quantitative reverse transcription-polymerase chain reaction (qRT-PCR) was used to validate the differentially expressed RNAs (DE-RNAs). In all 9,593 circRNAs and 20,030 mRNAs were found in the two groups of specimens, among which 50 DE-circRNAs and 244 DE-mRNAs were significantly upregulated, and 44 DE-circRNAs and 294 DE-mRNAs were significantly downregulated. Based on the regulatory mechanism of ceRNAs, circRNAs can directly bind microRNAs (miRNAs) to affect mRNA expression, and the expression trends of circRNAs and mRNAs are consistent. According to this mechanism, we constructed two ceRNA networks by using the RNA sequencing data. The function of these DE-circRNAs was further elucidated by enrichment analysis. In summary, the present study showed that the circRNA expression profile of BD patients is altered, and a ceRNA regulatory network was constructed, which provided a hypothesis about the pathogenesis of BD.

## Introduction

Bipolar disorder is a mood disorder disease of unknown etiology with a series of serious symptoms. the average age of onset is 30 years old (McIntyre et al., 2020). Evidence from genetic studies indicates that BD shows high heritability, and the associated genetic risk and gene expression profiles overlap (Shao and Vawter, 2008; Cardno and Owen, 2014). In particular, genetic and epigenetic changes can affect gene expression by directly modifying mRNA templates or by regulating post transcriptional translation, which plays an important role in the pathogenesis of BD. Non-coding RNAs (ncRNA), including microRNAs, tRNA, and circRNAs, involve in regulating gene expression and appear to be altered by genetic and environmental exposure in ways that are associated with mental illness, suggesting that they play a critical role in maintaining normal physiological function and homeostasis of the nervous system (Geaghan and Cairns, 2015; Mahmoudi and Cairns, 2017; Rusconi et al., 2020).

Circular RNA is a newly hot spot of endogenous ncRNA that is evolutionarily conserved in eukaryotic species and highly expressed in mammals (Jeck et al., 2013). CircRNAs are formed from special reverse complementary precursor mRNAs, which are cut at variable positions and cyclized head to tail. This special structure makes them more stable and not degraded by RNase R. CircRNAs are increasingly recognized as major epigenetic regulators. Their function is similar to that of a sponge of endogenous RNA or miRNA and can competitively inhibit the transcriptional regulation of miRNA. CircRNAs can also regulate variable splicing or transcription by inhibiting transcription initiation sites (Li et al., 2015), isolate RNA-binding proteins (RBPs) and form RNA-protein complexes affects the localization and transportation of RBPs and their related mRNAs (Fischer and Leung, 2017; Haque and Harries, 2017). Recently, some circRNAs were proven to be successfully encode functional proteins (Legnini et al., 2017). Increasing evidence shows that circRNAs are involved in the occurrence and progression of many human diseases, such as cardiovascular diseases, cancer, and central nervous system disorders, including schizophrenia (SZ), major depressive disorder (MDD), amyotrophic lateral sclerosis (ALS), and Alzheimer’s disease (AD) (Su et al., 2019; Jaé and Dimmeler, 2020; Mehta et al., 2020). The study also revealed the perturbation of ncRNAs in tissues and body fluids and suggested that they might serve as diagnostic biomarkers or therapeutic targets (Mehta et al., 2020). For example, 22 circRNAs were dysregulated in the peripheral blood samples of SZ patients. One of those circRNAs, circDPYD, was dysregulated in blood samples from coronary artery disease patients and was proven to increase TRPM3 expression via inhibiting miR-130a-3p (Pan et al., 2017). Interestingly, the parental gene of this circRNA is DPYD, which is located at a genome-wide significant risk locus for SZ. Similarly, a major alteration of circRNAs has been found in ALS patients, among which circ_0023919, circ_0063411, and circ_0088036 are considered candidate diagnostic biomarkers (Dolinar et al., 2019). The Cdr1 as contains more than 70 conserved miRNA target sites, which can sponge miR-7 and thus inhibit the expression of the ubiquitin carboxyl terminal hydrolase L1 (UCHL1) gene; this leads to decreased β-amyloid precursor protein (APP) and β-site APP cleaving enzyme 1 (BACE1) protein levels, indicating that ciRS-7 may be a potential therapeutic target for AD (Zhao et al., 2016; Shi et al., 2017). However, few reports on the circRNA-related ceRNA network in BD patients. Thus, the identification of BD-related circRNA profiles and the characterization of these circRNAs and their functions are urgent goals that will provide very useful information.

This research aimed to investigate differentially expressed circRNAs and mRNAs (DE-circRNAs and DE-mRNAs) in BD patients vs. controls and to comprehensively analyze the “DEcircRNA-miRNA-DEmRNA” ceRNA network involved in BD. The findings of this work may put forward some candidate targets for the development of novel treatment policies for BD.

## Materials and Methods

### Samples and RNA Isolation

From January 2019 to December 2019, according to the structured clinical interview of DSM-IV, two psychiatrists diagnosed 20 patients aged 19–27, and collected their blood samples and 20 normal people aged 19–25 as controls (basic clinical data are shown in Table 1). These collected blood samples were applied in two parts. Four blood samples from BD patients and four normal blood samples were used for high-throughput sequencing, and the remaining blood samples were used for subsequent qPCR to verify the sequencing results. Total RNA was isolated from peripheral blood samples using a miRVana^TM^ RNA isolation Kit according to the reagent instructions. RNA concentration and OD260/OD280 were tested with a NanoDrop 2000 instrument (Thermo Fisher Scientific, United States). RNA integrity was tested by agarose gel electrophoresis. This protocol was approved by the Ethics Committee of Jiangxi Mental Hospital, Nanchang, China. All participants gave informed consent for the collection and use of their samples for this study.

**TABLE 1:** Basic clinical data of BD and normal controls.

Variables	BD (*n* = 20)	NC (*n* = 20)	*P*
Age	23.8 ± 4.53	22.7 ± 3.5	0.921
Age range (years)	19–27	19–25	
Gender			
Male	12	10	
Female	8	10	
Education (years)	15.3 ± 4.0	14.87 ± 3.9	0.957
Duration of illness (months)	33.25 ± 12.07	N/A	
Family history [*n*(%)]	7 (35%)	N/A	
HAMD	34.38 ± 5.04	N/A	
YMRS	25.65 ± 13.22	N/A	

*NC, normal control; HAMD, Hamilton depression rating scale; YMRS, young manic rating scale; N/A, not available.*

**P* < 0.05 was considered statistically significant.*

### Illumina High-Throughput Sequencing

Ribosomal RNA was extracted using the TruSeq Stranded Total RNA with Ribo-Zero Gold following the reagent instructions. RNA libraries were constructed by using rRNA-depleted RNAs with the Illumina Library Prep Kit according to the reagent instructions. The constructed RNA library was qualified by Agilent 2100 Bioanalyzer and sequenced by Illumina sequencer (HiSeq X Ten) in PE150 mode following the manufacturer’s instructions. All sequencing raw reads subjected to quality control (Q40). Next, the reads of sequencing difference was cleared using Cutadapt software (V1.9.3). The clean reads were used for the analysis of circRNAs and mRNAs by using Hisat2 software (Kim et al., 2015) and the circBase database (Glažar et al., 2014). All data analyses were operated by Shanghai OE Biotech Co., Ltd. (China).

### Identification of DE-circRNAs and DE-mRNAs

The number of circRNA counts in each sample was standardized by using DEseq (Anders and Huber, 2012) software, the fold change (log_2_FC) was calculated, and the different significance testing of reads number was carried out by a negative binomial distribution test. HT-seq count software (Anders et al., 2015) was performed to obtain the number of mRNA reads in all samples, and Cufflinks software (Roberts et al., 2011) was used to calculate the fragments per kilobase per million (FPKM) values of the mRNAs. circRNA with *p*-values lower than 0.05 and log_2_fold change (FC) value higher than 1 were considered differentially expressed, while mRNA with *p*-values lower than 0.05 and log_2_fold change (FC) value higher than 0.58 were considered differentially expressed.

### qRT-PCR Validation of DE-circRNAs and DEmRNAs

Quantitative reverse transcription-polymerase chain reaction was performed using a PCR thermocycler (ABI 7900TH, United States) to validate DE-circRNAs and DE-mRNAs, which included three upregulated and three downregulated circRNAs and mRNAs. The circRNA-specific primer sequences were designed to span the reverse-spliced sequences of circular RNAs but not mRNA of the same sequence (The primer sequences are presented in Table 2). The expression levels of the DE-circRNAs and DE-mRNAs were standardization to (input the reference gene, e.g., β-actin) and were calculated via the 2^–ΔΔCt^ method (1) Δ Ct_*con*_ = mean CT value of target gene – mean CT value of internal reference gene Δ Ct_*BD*_ = mean CT value of target gene – mean CT value of internal reference gene, (2) ΔΔ Ct = Δ Ct_*BD*_- Δ Ct_*con*_, (3) Change Fold = 2^–ΔΔCt^ (con, normal control group; BD, BD group).

**TABLE 2:** List of RNA primer sequence verified by qPCR.

	Gene symbol	Forward primer	Reverse primer	Product length (bp)	Tm (°C)
1	IL1B	AAGAAACCCTCTGTCATTCG	GACACTGCTACTTCTTGCC	126	60
2	MAFB	TGCTGAGAGAGAGAACCGA	CTGTAGTCCAGAACACTCCT	96	60
3	GLUL	GGGAATTTCAGATTGGACCT	CAAAGTCTTCACACACACGA	93	60
4	ABO	CCATCAAGAAATACGTGGCT	TGGTCGGTGAAGACATAGTAG	100	60
5	SP6	GGGAAGGTGCGTATTTATTCAG	TACCGACCCAGTCAAATTCAT	117	60
6	TIMP3	GGTATCACCTGGGTTGTAACT	GAAATTGGAGAGCATGTCGG	104	60
7	Chr7:142060311_142086328_+	TCCAGCCATTTCTGGCAA	TGGGAAGGCCACATAAGC	199	60
8	Chr6:29945234_30009177_+	GATCCTGCCCTTGGTTTG	GCAGCTGTCTCACACTTTAC	223	60
9	Chr13:50027207_50045232_−	AGAGGGCAATAAATGCCAC	CTTCCTGGATACTCTCCTGTAG	162	60
10	Chr19:54222943_54280299_−	AGACAACCCCATGACAAGAA	GATGGTCCCTGTCTGCAC	199	60
11	Chr5:155854549_155870424_+	TATCTATTGAAGCTGGGAGGGT	CCAACAAGTTGAATCAGCATAA	104	60
12	Chr13:40826506_40855815_−	GATGTATTCAACAGTCCACCTC	CTTGATAGTGGTTTGGATGCTT	103	60
13	ACTB	CATTCCAAATATGAGATGCGTT	TACACGAAAGCAATGCTATCAC	133	60

### Gene Ontology and Pathway Enrichment Analyses

The functional annotation of the target mRNAs of DE-circRNAs was performed by Gene Ontology (GO)^1^ term and Kyoto Encyclopedia of Genes and Genomes (KEGG)^2^ pathway analyses. Corrected *P*-values < 0.05 were considered to indicate significant enrichment.

### Construction of “DE-circRNA-miRNA-DE-mRNA” ceRNA Networks

DE-circRNA-miRNA binding were predicted using StarBase (V2.0). The interaction between miRNA and target mRNAs was conducted with miRanda^3^ and TargetScan^4^ software, respectively. The circRNA-miRNA-mRNA network was visualized by using Cytoscape v3.7.1 software^5^.

### Statistical Analysis

The obtained statistical data were presented as the mean ± SEM by using SPSS 23.0 and were compared via Student’s *t*-test. A two-tailed *P* < 0.05 was considered statistical significance.

## Results

### Distribution Profiles of circRNAs

**FIGURE 1:**
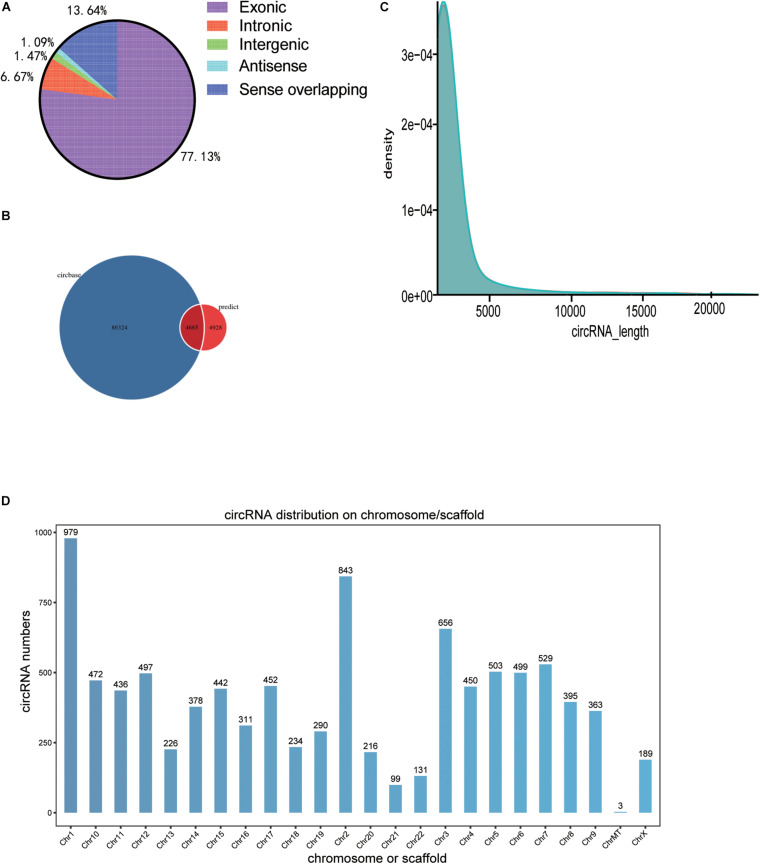
Distribution spectrum of circRNAs. **(A)** Map of the circRNA class distribution, **(B)** Classification of circRNA based on the circBase database. **(C)** Length density map of circRNAs. **(D)** The histogram represents the total cirRNAs distribution of the test sample, and the ordinate represents the number of transcriptional parent genes of the number of cirRNAs on each chromosome Chr, chromosome.

A total of 9,593 circRNAs were detected between the normal control and BD groups by RNA sequencing. According to the position of circRNA in their parental gene, it can be divided into different types, as shown in Figure 1A. A total of 4,665 circular RNAs (48.6%) were identified in the circBase database from previous studies, while 4,928 (51.4%) circRNAs were not identified in the database (Figure 1B). Most of 9,593 circular RNAs in the two groups ranged in length from 100 to 3,500 BP (Figure 1C). The histogram represents the total cirRNAs distribution of the test sample, and the ordinate represents the number of transcriptional parent genes of the number of cirRNAs on each chromosome (Figure 1D).

### Identification of DE-circRNAs in BD

**FIGURE 2:**
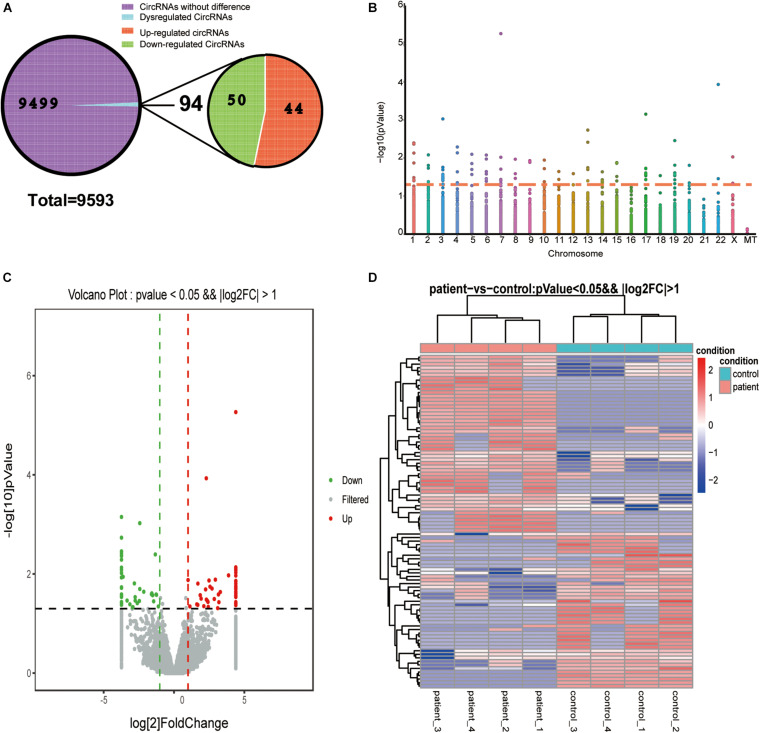
Differentially expressed circRNAs. **(A)** Pie chart of circRNA expression classification. A total of 9,593 circRNAs were detected, of which 94 circRNAs were differentially regulated in the control and BD groups, containing 44 upregulated and 50 downregulated circRNAs. **(B)** Manhattan plot of DE-circRNAs across all chromosomes. Dots above the orange line represent DE-circRNAs. **(C)** Volcano plot of DE-circRNAs. Gray dots represent these circRNA that were not considerably differentially expressed. Green and red dots indicate significantly downregulated and upregulated DE-circRNAs, respectively. **(D)** Heat map of dysregulated DE-circRNAs. Rows and columns represent the genes and samples. The thresholds were a | log2FC| ≥ 1 and *P*-value ≤ 0.05.

We used DEG-seq software to analyze the DE-circRNAs between the BD and control groups. A total of 94 DE-circRNAs were obtained according to the significance thresholds of a | log2FC| ≥ 1 and a *P*-value ≤ 0.05, including 50 upregulated and 44 downregulated circRNAs (Figure 2A). A Manhattan plot shows the distribution of DE-circRNAs on chromosomes, and the threshold *P*-value ≤ 0.05 is converted it into log value for display, but not FC value. We found that DE-circRNAs were distributed in all chromosomes except chromosome 16 and 21, but not in mitochondria (MT) (Figure 2B). Furthermore, volcano plot and heat map were presented to identify DE-circRNAs between the two groups (Figures 2C,D).

### Validation of circRNA Expression by Quantitative Real-Time PCR (qRT-PCR)

**FIGURE 3:**
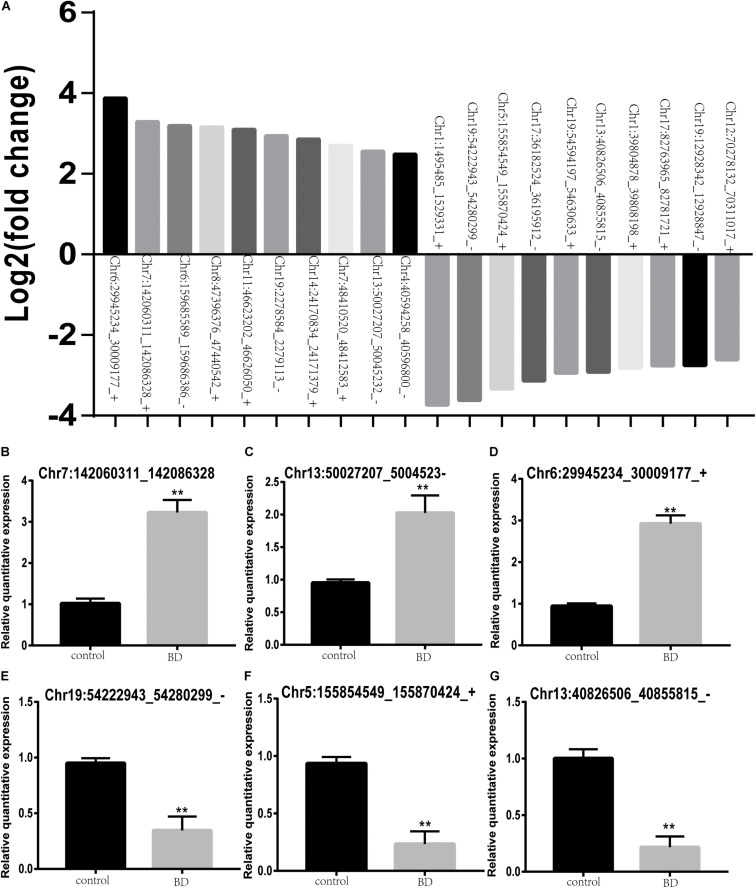
Validation of the expression levels of DE-circRNAs in BD patients and control subjects. **(A)** Top 10 upregulated and downregulated DE-circRNAs between two groups. **(B–G)** Chr7:142060311_142086328 **(B)**, Chr13:50027207_50045232_– **(C)**, and Chr6:29945234_30009177_+ **(D)** were the three upregulated circRNAs. Chr19:54222943_54280299_– **(E)**, Chr5:155854549_155870424_+ **(F)** and Chr13:40826506_40855815_– **(G)** were downregulated circRNAs. Black stars indicate significant differences.

To validate the expression profiles of those DE-circRNAs, we selected three upregulated and three downregulated circRNAs from the top 10 upregulated DE-circRNAs and the top 10 downregulated DE-circRNAs (Figure 3A). As shown in Figures 3B–G, Chr7:142060311_142086328, Chr13:50027207_50045232_− and Chr6:29945234_30009177_+ were the three selected upregulated circRNAs. Chr19:54222943_54280299_−, Chr5:155854549_155870424_+ and Chr13:40826506_40855815_− were the three selected downregulated circRNAs. These results were consistent with the transcriptional sequencing data.

### Target miRNAs of DE-circRNAs

**FIGURE 4:**
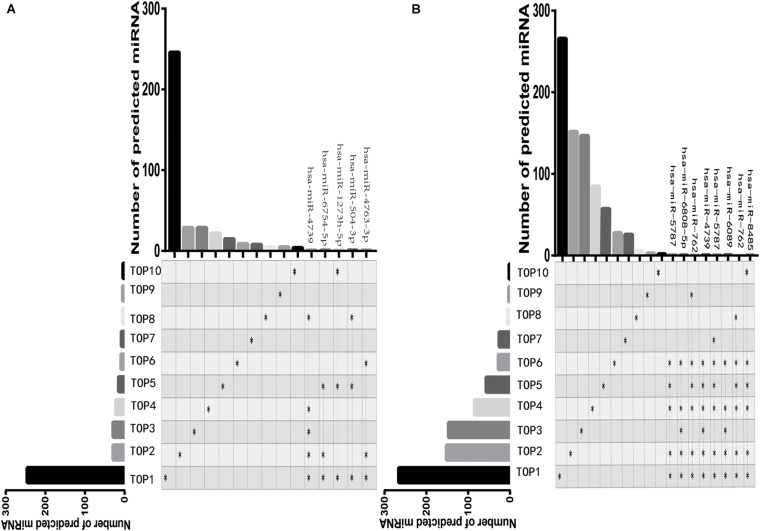
The visualization map of DE-circRNAs target miRNAs. The composite matrix graph shown visualize set overlap. The vertical axis presents the number of target miRNAs of each DE-circRNA. The black star on the left side of the matrix and the corresponding column above represent the number of miRNAs predicted by the differentially top10 expressed circRNA. The blue line and the stars on the right represent that the target miRNA predicted by the top10 DE-circRNA overlapped at least three times among different groups. Top1-top10 represent the top ten DE-circRNAs. A, The visualization map of the top 10 upregulated DE-circRNAs. B, The visualization map of the top 10 downregulated DE-circRNAs.

Next, we respectively, predicted the 362 target miRNAs of the top 10 upregulated DE-circRNAs and the 772 target miRNAs of the top 10 downregulated DE-circRNAs via StarBase (v2.0), Miranda (v3.3a), TargetScan (v7.0), and miRTarBase (v6.1) (Figure 4A,B). Among these miRNAs, five miRNAs (hsa-miR-4739, hsa-miR-6754-5p, hsa-miR-1273 h-5p, hsa-miR-504-3p, and hsa-miR-4763-3p) were predicted targets of not less than three of the top 10 upregulated DE-circRNAs (Figure 4A), and eight miRNAs (hsa-miR-5787, hsa-miR-6808-5p, hsa-miR-762, hsa-miR-4739, hsa-miR-5787, hsa-miR-6089, hsa-miR-762, and hsa-miR-8485) were predicted targets of not less than five of the top 10 downregulated DE-circRNAs (Figure 4B). The top 10 upregulated DE-circRNAs, top 10 downregulated DE-circRNAs and their predicted miRNAs were used for subsequent target mRNA prediction.

### Identification and Validation of DE-mRNAs in BD

**FIGURE 5:**
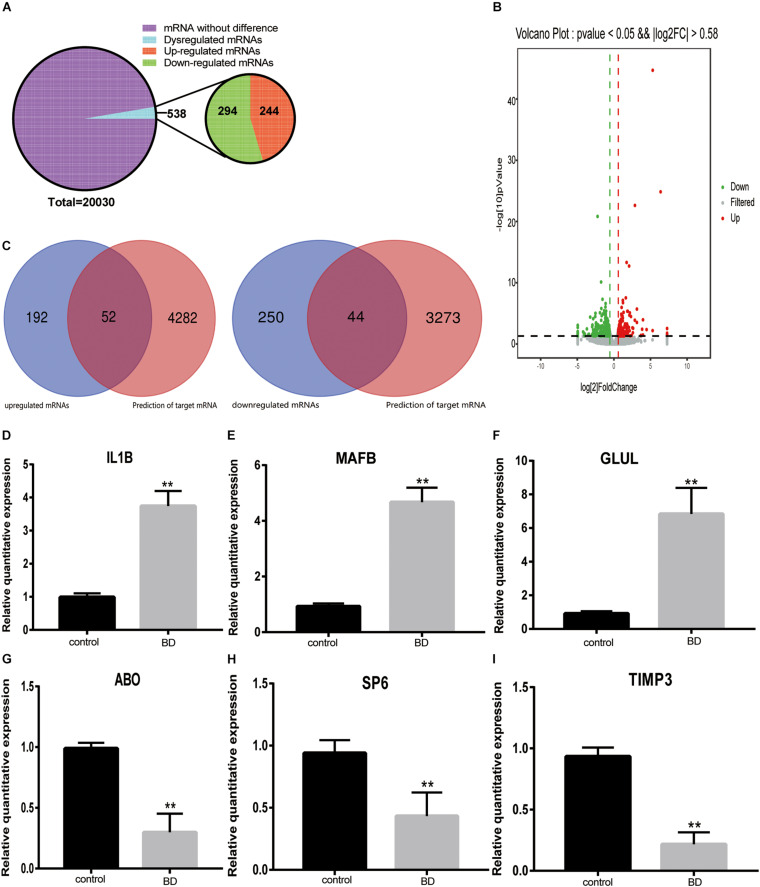
The mRNAs expression pattern and validation of DE-mRNA expression. **(A)** Pie chart of mRNAs expression classification. All 20,030 mRNAs were detected in silicon. A total of 538 mRNAs were dysregulated between two groups, including 244 upregulated and 294 downregulated circRNAs. **(B)** Volcano plot of mRNAs. Gray dots represent these mRNAs that were not considerably differentially expressed. Green and red dots indicate significantly downregulated and upregulated DE-RNAs, respectively. The thresholds were a | log2FC| ≥ 0.58 and *P*-value ≤ 0.05. **(C)** Venn diagram of the intersection of sequenced mRNAs and predicted mRNAs. The number of overlapping mRNAs between the upregulated DE-mRNAs determined by RNA-seq and the mRNAs predicted according to the top 10 upregulated circRNAs was 52. The number of overlapping mRNAs between the downregulated DE-mRNAs determined by RNA-seq and the mRNAs predicted according to the top 10 downregulated circRNAs was 44. **(D–I)** qRT-PCR was used to validate six mRNAs in the two groups. Three upregulated DE-mRNAs: IL1B **(D)**, MAFB **(E)** and GLUL **(F)**. Three downregulated DE-mRNAs: ABO **(G)**, SP6 **(H)** and TIMP3 **(I)**. **Indicate significant differences.

All transcriptional sequencing identified 20,030 mRNAs in the control and BD groups. A total of 538 significantly DE-mRNAs were obtained according to the thresholds of a | log_2_FC| ≥ 0.58 and a *P* ≤ 0.05, among those 244 were upregulated DE-mRNAs and 294 were downregulated DE-mRNAs (Figure 5A). We generated a volcano plot of the DEGs between the two groups (Figure 5B). In the above results, we predicted 362 miRNAs of upregulated TOP 10 cirRNAs, based on which we further predicted 4,334 mRNAs. We also predicted 772 miRNAs of downregulated TOP 10 cirRNAs, based on which predicted 3317 mRNAs. We intersected the mRNA predicted by the upregulated and downregulated top 10 cirRNAs with the differential genes in our own mRNA sequencing data. Finally, we obtained obtain 52 upregulated and 44 downregulated mRNAs (Figure 5C). Among these mRNAs, we selected three upregulated and three downregulated mRNAs that we were interested to valid their expression profile. As shown in Figures 5D–I, IL1B, MAFB, and GLUL were the three selected upregulated mRNAs. While ABO, SP6, and TIMP3 were the three selected downregulated mRNAs.

### Functional Enrichment Analysis

**FIGURE 6:**
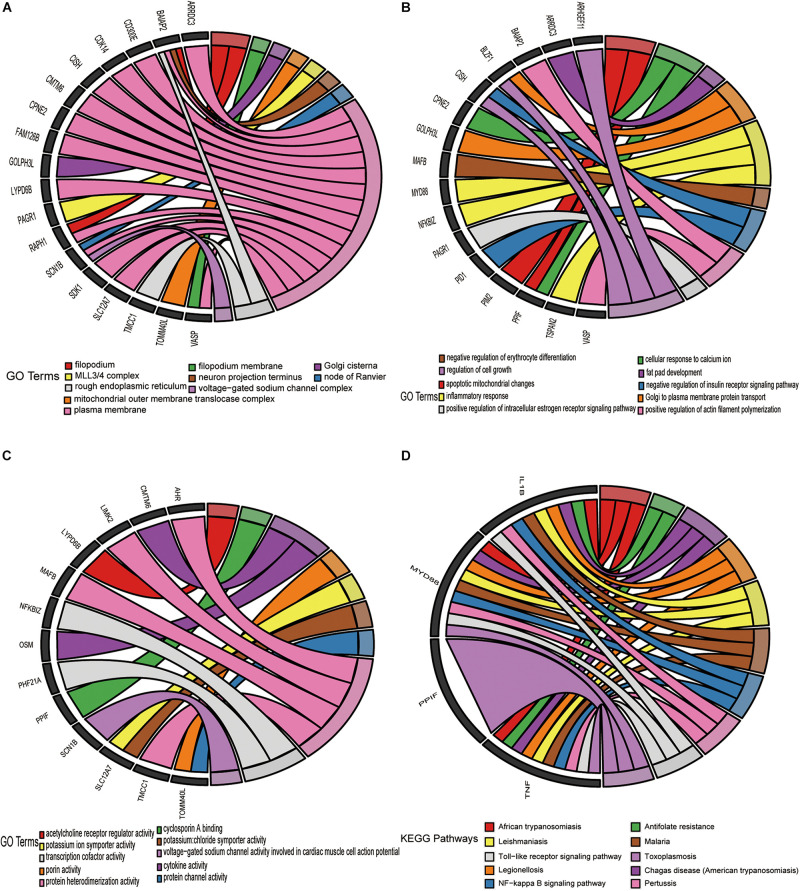
Enrichment analysis of 52 upregulated target DE-mRNAs. Chord plots was used to visualize the enrichment analysis of these DE-mRNAs. **(A)** GO item cellular CC analysis. **(B)** GO item Bp analysis. **(C)** GO item MF analysis. **(D)** KEGG pathway analysis. The thresholds were a | log2FC| ≥ 0.58 and *P*-value ≤ 0.05.

**FIGURE 7:**
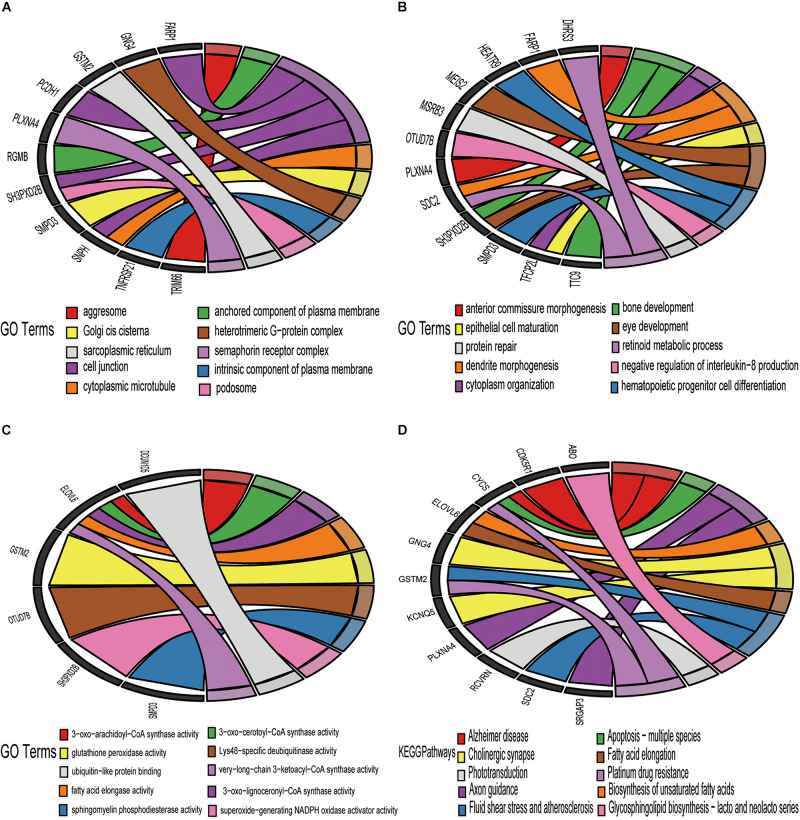
Enrichment analysis of 44 downregulated target DE-mRNAs. Chord plots was used to visualize the enrichment analysis of these DE-mRNAs. **(A)** GO item cellular CC analysis. **(B)** GO item Bp analysis. **(C)** GO item MF analysis. **(D)** KEGG pathway analysis. The thresholds were a | log2FC| ≥ 0.58 and *P*-value ≤ 0.05.

Gene Ontology and Kyoto Encyclopedia of Genes and Genomes pathway functional enrichment analyses were executed derived from the 52 overlapping upregulated and 44 overlapping downregulated mRNAs in Figure 5C. These overlapping upregulated mRNAs were mainly associated with the following terms: plasma membrane, filopodium, and rough endoplasmic reticulum, in the cellular component (CC) category (Figure 6A); regulation of cell growth, inflammatory response, and apoptotic mitochondrial changes in the biological process (BP) category (Figure 6B); and protein heterodimerization activity, transcription cofactor activity, and cytokine activity in the molecular function (MF) category (Figure 6C). These overlapping downregulated mRNAs were mainly enriched in the following terms: semaphoring receptor complex in the CC category (Figure 7A); negative regulation of interleukin-8 production in the BP category (Figure 7B); and superoxide-generating NADPH oxidase activator activity in the MF category (Figure 7C). KEGG pathway enrichment analysis showed that these upregulated genes were mainly associated with the NF-kappa B signaling pathway, the Toll-like receptor signaling pathway and antifolate resistance (Figure 6D), while the downregulated genes were mainly associated with glycosphingolipid biosynthesis-lacto, cholinergic synapse and Alzheimer’s disease (Figure 7D).

### Visual Graph of ceRNA Networks

**FIGURE 8:**
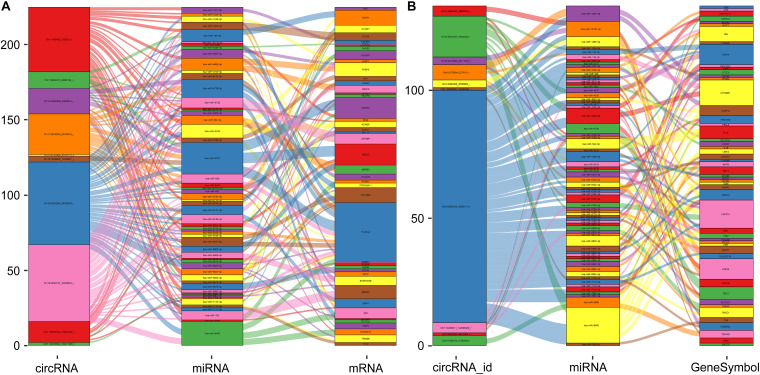
The visualization chart of ceRNA networks. **(A)** The ceRNA regulatory network of the top 10 downregulated DE-circRNAs. **(B)** The ceRNA regulatory network of the top 10 upregulated DE-circRNAs. The rectangle represents genes, and the size of the rectangle represents the number of gene connections.

Based on the regulatory mechanism of ceRNAs, circRNAs, and mRNAs can competitively bind to miRNA binding sites and act as ceRNAs. Moreover, the expression trends of circRNAs and mRNAs are consistent. Therefore, we established two ceRNA regulatory networks of DE- circRNAs and DE-mRNAs. As shown in Figure 8A, the ceRNA network included the top 10 downregulated DE-circRNAs, 44 downregulated DE-mRNAs (Figure 5C) and 56 miRNAs predicted by both. The ceRNA regulatory network presented in Figure 8B comprises the top 10 upregulated DE-circRNAs, 52 upregulated DE-mRNAs (Figure 5C) and 68 miRNAs predicted by both.

## Discussion

The incidence of BD, which seriously affects human health and quality of life, is gradually increasing (McIntyre et al., 2020). Although some research progress has been acquired recently, the etiopathogenesis of BD have not yet been complete elucidated. The development of bioinformatics has accelerate the progress of disease mechanisms and treatment strategy (Zhang et al., 2017). In this work, we comprehensively analyzed circRNA and mRNA expression pattern of BD patients and constructed circRNA-associated ceRNA network.

Circular RNAs are involved in the adjustment of neuronal activity, plasticity, depolarization, and synaptic transmission, which are key steps involved in the pathophysiology of mental diseases (Piwecka et al., 2017; Mahmoudi et al., 2020). Recently, Ebrahim et al., identified 22 and 33 circRNAs showing significant changes in peripheral blood mononuclear cells from SZ and BD patients, respectively, compared to normal controls (Mahmoudi et al., 2021). Although these findings were validated by qPCR in a large sample, we were unable to replicate these results in our cohort, likely because of the different sample sizes. In addition, different sequencing platforms may lead to inconsistent observations. Moreover, these previous authors did not establish the ceRNA regulation mechanism of the DE-circRNAs. Therefore, the expression and function of circRNAs in BD patients are in need of further study. In this research, we implemented an analysis of DE-circRNAs and DE-mRNAs between BD patients and healthy controls and constructed circRNA-associated ceRNA network.

We gained 538 DE-mRNAs and 94 DE-circRNAs between the two groups. Based on the ceRNA theory, circRNAs and mRNAs serve as ceRNAs, and they share the same miRNA binding sites. We constructed two ceRNA regulatory networks. The network of the top 10 upregulated ceRNAs was composed of 52 DE-mRNAs, 10 DE-circRNAs, and 68 target miRNAs and included 134 circRNA-miRNA-mRNA ceRNA circular pathways. The network of the top 10 downregulated ceRNAs was composed of 44 DE-mRNAs, 10 DE-circRNAs, and 56 target miRNAs and included 226 circRNA-miRNA-mRNA ceRNA circular pathways.

To assess the functions of the differentially expressed genes (DEG), GO, and KEGG signaling pathway analyses were executed derived from the 52 overlapping upregulated mRNAs and 44 overlapping downregulated mRNAs. We found that these DE-mRNAs were mainly involved in the regulation of cell growth, immune imbalance, inflammatory response and mitochondrial apoptosis. It has long been reported that BD is a chronic disabling medical disease associated with immune imbalance (Barbosa et al., 2014). During manic or depressive episodes in BD patients, activated T cell counts increase, regulatory T cell counts decrease, and plasma proinflammatory cytokine levels increase. The increased levels of inflammatory cytokines may be involved in the occurrence and development of diseases by regulating neuronal metabolism, synaptic plasticity, and activation of hypothalamic pituitary adrenal (HPA) axis (Raison et al., 2006; Miller et al., 2009). Although the mechanism responsible for the immune disorder is still unclear to a large extent, recent studies have shown that the signaling cascade of innate and adaptive cells is altered (Qian and Cao, 2013). In BD patients, the activity of some signaling pathways involved in immune response increased, such as NF-κB and MAPK signaling pathway (Barbosa et al., 2013). In addition, increases in the NLRP3 inflammasome and caspase-1 levels are associated with increases in serum IL-1B and IL-18 levels in patients with major depression. Toll-like receptor (TLR) family is significant mediators of the inflammatory response that connect innate immunity and acquired immunity. TLR involves a complex intracellular cascade including MyD88 (the common adaptor protein of all TLRs), IRAK, and TRAF family members (Majewska and Szczepanik, 2003; Akira and Takeda, 2004; Ishii et al., 2005). Therefore, immune imbalances and the inflammatory response may be important pathogenic mechanisms of BD. The results of Go and KEGG pathway analysis showed that the DEG in ceRNA network was mainly related to immune imbalance and inflammatory response, which may be the main pathogenesis of BD, providing an important reference for the follow-up study of BD pathogenesis.

Some strengths and limitations of this study should be acknowledged. First, we identified DE-circRNAs and DE-mRNAs in BD patients versus control subjects and comprehensively analyzed a ceRNA network involved in BD. Second, we used bioinformatics technology, which can aid in understanding the genes and networks that are responsible for diseases. Due to the limitation of bioinformatics analysis and the small number of samples, especially in BD group, the statistical ability of microarray analysis is limited. Therefore, more *in vivo* and *in vitro* experiments are needed to verify the results of bioinformatics.

## Conclusion

Our study evaluated circRNA expression in the peripheral blood of BD patients and assessed the potential functions of DE-circRNAs via bioinformatics. Moreover, we created two DE-circRNA related regulatory networks based on the ceRNA theory. In the networks, one circRNA can indirectly regulate multiple mRNAs expression via binding to multiple miRNAs, suggesting that circRNAs may be related to the intricate mechanism of the development of BD.

## Data Availability Statement

The datasets presented in this study can be found in online repositories. The names of the repository/repositories and accession number(s) can be found here: NCBI SRA PRJNA735880.

## Ethics Statement

The studies involving human participants were reviewed and approved by the Ethics Committee of Jiangxi Mental Hospital. The patients/participants provided their written informed consent to participate in this study.

## Author Contributions

LH and BW designed the subject. YF wrote the manuscript. CZ, XF, and QW collected and visualized the results. All authors contributed to the article and approved the submitted version.

## Conflict of Interest

The authors declare that the research was conducted in the absence of any commercial or financial relationships that could be construed as a potential conflict of interest.

## Publisher’s Note

All claims expressed in this article are solely those of the authors and do not necessarily represent those of their affiliated organizations, or those of the publisher, the editors and the reviewers. Any product that may be evaluated in this article, or claim that may be made by its manufacturer, is not guaranteed or endorsed by the publisher.

## Footnotes

1
http://www.geneontology.org

2
www.kegg.jp/kegg/kegg1.html

3
http://www.mirdb.org/

4
http://www.targetscan.org/vert_71/

5
https://cytoscape.org/
